# Evaluating a motor progression connectivity model across Parkinson’s disease stages

**DOI:** 10.1007/s00415-024-12703-8

**Published:** 2024-10-07

**Authors:** Mallory L. Hacker, David A. Isaacs, Nanditha Rajamani, Kian Pazira, Eli Abdou, Sheffield Sharp, Thomas L. Davis, Peter Hedera, Fenna T. Phibbs, David Charles, Andreas Horn

**Affiliations:** 1 Department of Neurology, Vanderbilt University Medical Center, Nashville, TN USA; 2 Movement Disorder and Neuromodulation Unit, Department of Neurology, Charité, Universitätsmedizin Berlin, corporate member of Freie Universität Berlin and Humboldt- Universität Zu Berlin, Berlin, Germany; 3 Center for Brain Circuit Therapeutics Department of Neurology Brigham and Women’s Hospital, Harvard Medical School, Boston, MA USA; 4 Department of Neurology, University of Louisville, Louisville, KY USA; 5 Department of Neurosurgery and Center for Neurotechnology and Neurorecovery, Massachusetts General Hospital, Boston, MA USA

**Keywords:** Parkinson’s disease, Deep brain stimulation, Movement disorders, Subthalamic nucleus, Motor symptoms

## Abstract

**Background:**

Stimulation of a specific site in the dorsolateral subthalamic nucleus (STN) was recently associated with slower motor progression in Parkinson’s Disease (PD), based on the deep brain stimulation (DBS) in early-stage PD pilot clinical trial. Here, subject-level visualizations are presented of this early-stage PD dataset to further describe the relationship between active contacts and motor progression. This study also evaluates whether stimulation of the sweet spot and connectivity model associated with slower motor progression is also associated with improvements in long-term motor outcomes in patients with advanced-stage PD.

**Methods:**

Active contacts of the early-stage PD cohort (N = 14) were analyzed alongside the degree of two-year motor progression. Sweet spot and connectivity models derived from the early-stage PD cohort were then used to determine how well they can estimate the variance in long-term motor outcomes in an independent STN-DBS cohort of advanced-stage PD patients (N = 29).

**Results:**

In early-stage PD, proximity of stimulation to the dorsolateral STN was associated with slower motor progression. In advanced-stage PD, stimulation proximity to the early PD connectivity model and sweet spot were associated with better long-term motor outcomes (R = 0.60, P < 0.001; R = 0.37, P = 0.046, respectively).

**Conclusions:**

Results suggest stimulation of a specific site in the dorsolateral STN is associated with both slower motor progression and long-term motor improvements in PD.

## Introduction

Recently, we demonstrated that deep brain stimulation (DBS) of a specific site within the dorsolateral region of the subthalamic nucleus (STN) is associated with slower motor progression for early-stage Parkinson’s disease (PD) patients [1]. This site receives cortical (hyperdirect) input from the primary motor (M1) and supplementary motor area (SMA) but not from the pre-SMA. These results built upon a post hoc analysis of the DBS in early-stage PD pilot clinical trial [2]. The specific site that was identified in early-stage PD is similar to, yet slightly more ventral than, a previously reported metanalytic location associated with optimal symptomatic motor benefit in advanced-stage PD [3]. Moreover, a similar connectivity profile (i.e., stimulating M1/SMA) has also been associated with symptomatic motor improvement in advanced-stage PD [4–6]. Therefore, it could be that these sites, i.e., the one associated with slower motor progression and the one associated with optimal motor symptom improvements, are the same. However, such a relationship has not yet been empirically analyzed.

Our early-stage PD electrode localization study applied DBS sweet spot mapping [7] and DBS fiber filtering [8] to evaluate relationships between motor progression and stimulation location across the entire early-stage PD DBS cohort [1]. This led to a specific site (sweet spot) and a set of connections associated with slower motor progression (connectivity model). Here, we describe the relationship between active contacts and slower motor progression further by presenting subject-level visualizations of this early DBS dataset. We also evaluate whether the sweet spot and connectivity models constructed from the DBS in early-stage PD pilot clinical trial cohort are associated with long-term motor improvements in an independent cohort of advanced-stage PD patients treated with DBS per standard of care.

## Methods

### Cohorts

This study revisits data from the Vanderbilt “DBS in early-stage PD” pilot clinical trial (NCT00282152; IDEG050016; IRB#040797), which was a prospective, randomized, single-blind clinical trial evaluating bilateral STN-DBS in patients with early-stage PD (aged 50–75 years; PD medication duration 1–4 years; no history or evidence of dyskinesia or motor fluctuations) [9]. All 14 subjects with complete data for the prior electrode localization study [1] are included in this analysis, alongside clinical outcomes for subjects randomized to optimal drug therapy (ODT) in the pilot trial [9]. The present study also includes an independent cohort of 29 advanced-stage PD patients who received STN-DBS as standard care and participated in a long-term outcomes study at Vanderbilt University Medical Center (IRB#181198). The standard of care DBS subjects were recruited at least two years post-surgery. All subjects provided written informed consent for participation.

### Clinical outcomes

In the early-stage PD cohort, motor progression was defined as the change in the Unified Parkinson’s Disease Rating Scale part III score (UPDRS-III) from pre-operative baseline to 24 months measured after a one-week therapeutic washout (baseline: OFF medications; 24 months: OFF medications and OFF stimulation), which was blindly rated from video-recorded motor examinations at the conclusion of the trial [9]. In the advanced-stage PD cohort, pre-operative (ON medications) and longitudinal post-operative (ON medications, ON stimulation) UPDRS-III motor examinations were video-recorded and blindly rated. Long-term motor symptom changes were defined as the percent change from the pre-operative UPDRS-III to the longitudinal post-operative UPDRS-III. UPDRS-III scores for both cohorts do not include rigidity which cannot be evaluated via video recording. Levodopa equivalent daily doses (LEDDs) were calculated for both cohorts as previously described [10].

### Electrode localizations

Electrodes for both cohorts were localized with Lead-DBS [11, 12] using pre-operative T1-weighted and T2-weighted magnetic resonance (MRI) scans and post-operative computed tomography (CT) scans. The same processing pipeline for localizations previously reported for the early DBS cohort [1] was applied to the standard of care DBS cohort. Group visualizations were performed using Lead-Group [13].

### Estimation of stimulation volumes and validation of the sweet spot and motor progression models

The motor progression sweet spot and connectivity models were defined using the early-stage PD cohort as previously published [1]. Critically, both models were used exactly as previously published with all model parameters unchanged [1].

Sweet spot model: model definition (as carried out in [1]): the sweet spot identified in the DBS in early-stage PD pilot clinical trial was defined by voxels covering at least 3 electric fields (i.e., E-fields) with a vector magnitude > 0.2 V/mm [1]). Model validation (present study): we tested whether this published sweet spot would be able to account for variance in long-term clinical outcomes in a common advanced-stage PD cohort. To do so, E-field magnitudes for each patient in the standard of care, advanced-stage PD cohort were spatially rank-correlated with the sweet spot following published methods [14, 15]. This led to a Spearman correlation coefficient for each E-field, denoting how strongly it overlapped with the sweet spot in an interval between -1 and 1. These coefficients were then correlated with clinical improvements across the cohort [12]*.*

Optimal connectivity model: the same concept was applied to the a priori published connectivity model. Model definition (as carried out in [1]): the optimal connectivity model was identified using the DBS in early-stage PD pilot clinical trial cohort with the outcome of two-year motor progression (change from baseline to 24 months on examiner-blinded UPDRS-III score measured after a one-week therapeutic washout). Structural tract definitions of this model were based on an extended version of the DBS Tractography Atlas [16, 17], which was built using manual tractography results that were based on the HCP 1065 template provided with the DSI Studio software [18]. The HCP 1065 template was constructed from a total of 1065 subjects’ diffusion MRI data from the Human Connectome Project (2017 Q4, 1200-subject release). The cohort consisted of 575 females and 490 males, with ages ranging from 22 to 37 years (mean = 29; Q1 = 26, median = 29, Q3 = 32). Each of these streamlines were assigned a ‘Fiber-R-Score’ by correlating peak E-field magnitudes along the streamline with motor progression scores across the early DBS cohort. Model validation (present study): E-field magnitude values of the advanced-stage PD cohort were superimposed with the a priori published tract model [1] and average magnitudes were multiplied with the correlation weight of each streamline they intersected following the approach of [14, 15]. Resulting coefficients (‘Weighted Means of Fiber R-scores’) were then correlated with clinical improvements across the advanced-stage PD cohort.

### Data availability

The de-identified data from the standard of care DBS cohort will be made available upon reasonable request. The de-identified data and related study documents from the ‘DBS in early-stage PD’ pilot clinical trial are not being publicly shared at this time as they are currently being used for the development of a proprietary, multicenter, phase III, pivotal clinical trial (IDE G050016).

## Results

### DBS in early-stage PD active contacts grouped by motor progression thresholds

**Fig. 1:**
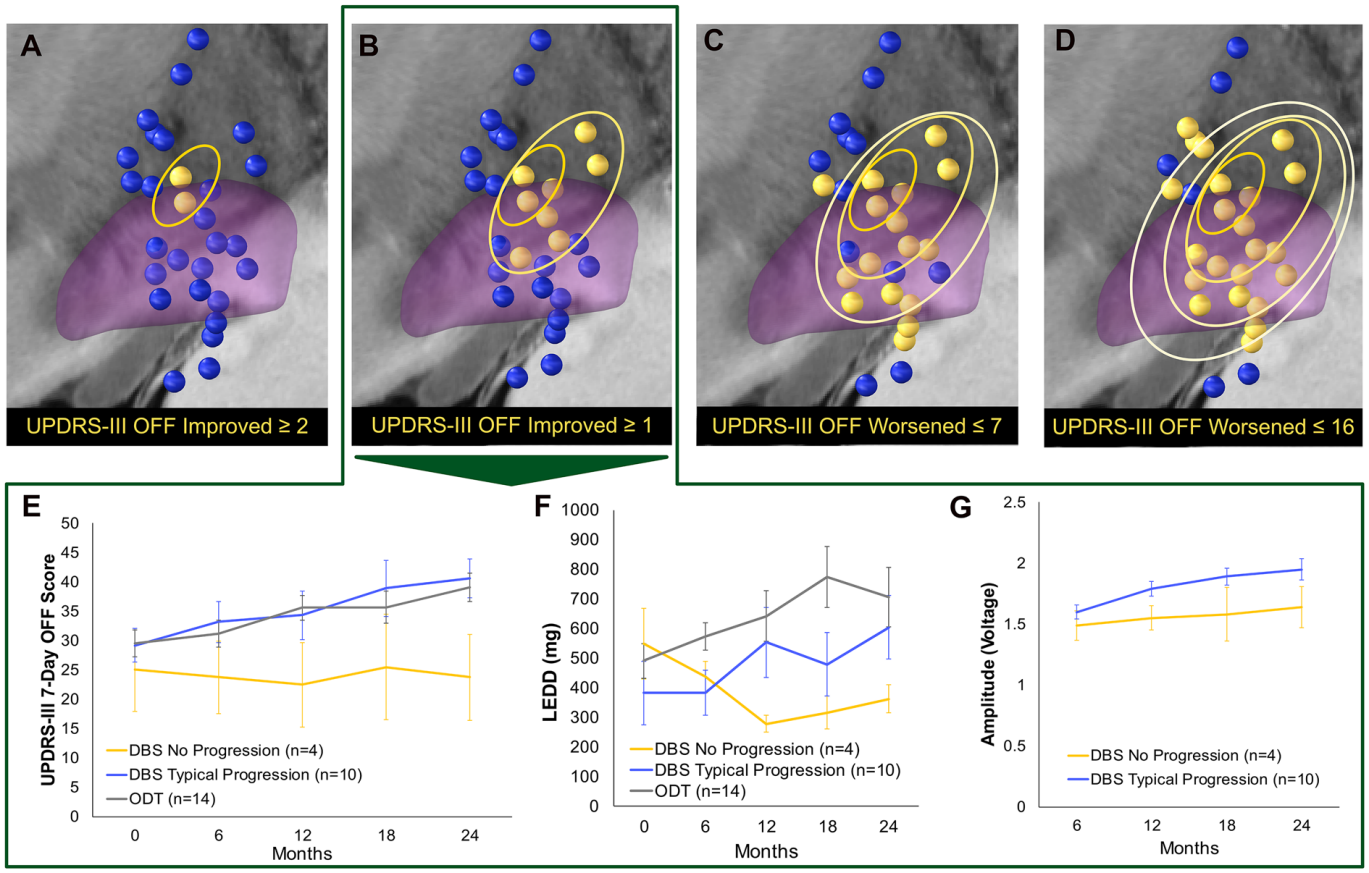
Active contacts vs motor progression in the DBS in early-stage PD pilot clinical trial cohort. **A**–**D** Mirrored active contacts for the early DBS cohort are shown when dividing the cohort into two groups (yellow spheres = top responders; blue spheres = remaining subjects) at several motor progression thresholds (reported below each respective panel). **E**–**G** Clinical features of DBS subjects without motor progression whose active contacts are shown in panel B (yellow lines) vs the other DBS subjects (blue lines) and subjects randomized to the optimal drug therapy (ODT) control group (gray lines). Early DBS subjects without motor progression (E, yellow lines) required fewer PD medications (F) and lower stimulation amplitudes (G) Mean ± SEM

Our prior electrode localization study used voxel-wise DBS sweet spot mapping and DBS fiber filtering across the entire cohort of patients from the DBS in early-stage PD pilot clinical trial to evaluate the relationships between motor progression and stimulation location [1]. The early-stage PD cohort (13/14 male; mean disease duration 2.6 ± 1.9 years; 60.9 ± 6.9 years old) was randomized to surgery as part of the ‘DBS in early-stage PD’ pilot clinical trial [9] (Table 1). The early-stage PD cohort was stimulated using an amplitude of 1.9 ± 0.3 V, with a 60 µsecond pulse width and a frequency of 130 Hz. In the present study, we visualize active contacts at the patient level for the 14 early DBS patients, who were divided into two groups (top responding patients and the remaining patients) to provide new insights into individual responses. Since no objective rationale could define the threshold used for grouping, this was repeated at various thresholds. The active contact for the top responding subject localized to the dorsolateral STN (Fig. 1A). This patient’s UPDRS-III score in the DBS OFF and medication OFF state did not deteriorate two years after surgery. In fact, it became two points better. When lowering the threshold to include all subjects with stable two-year UPDRS-III OFF scores (n = 4; UPDRS-III OFF score from baseline to 24 months improved by 1.3 ± 0.5 points) into the group of ‘top responders’, their stimulation sites showed proximity around the same coordinate within the dorsolateral STN (Fig. 1B). Lowering the threshold further included subjects with increasingly more motor progression and showed increasing distance of active contacts to this stimulation site (Fig. 1C, D). Figure 1E–G shows motor progression, change in LEDD, and stimulation amplitude for the four top responding early DBS subjects featured in Fig. 1B alongside the remaining early DBS subjects and the subjects randomized to ODT in the clinical trial. While no statistical comparisons were conducted due to the small sample sizes of the groups, the visualization of changes in motor progression revealed that early DBS subjects with active contacts outside of the dorsolateral STN (i.e., ‘typical responders’) appeared to follow a similar course to that of the ODT control group (Fig. 1E). Although these data do not support definitive conclusions, they suggest a similar trajectory of motor progression – on average – between typical responders and ODT control subjects. Additionally, early DBS subjects with active contacts close to the dorsolateral STN site required less medication on average at each follow-up visit as compared to baseline (Fig. 1F) and their mean stimulation voltages were lower than the typical DBS responders (Fig. 1G).

**Table 1:** STN-DBS subject characteristics

	Early-stage PD	Advanced-stage PD
N =	14	29
Sex, male: female	13:1	25:4
Age (years)	60.9 ± 6.9	60.9 ± 9.1
Disease duration (years)	2.6 ± 1.9	9.9 ± 5.0
LEDD (mg)		
Before surgery	430 ± 314	1378 ± 583
After surgery	534 ± 307^#^	748 ± 516^^^
Stimulation amplitude	1.9 ± 0.3 V^#^	2.9 ± 0.9 V^^^ (n = 21)
		3.0 ± 0.6 mA^^^ (n = 8)
UPDRS-III ON^+^		
Before surgery	23.1 ± 12.6	20.6 ± 9.3
After surgery	22.9 ± 12.6^#^	23.6 ± 8.9^^^
UPDRS-III OFF (one-week)^+^		
Before surgery	28.0 ± 10.2	N/A
After surgery	35.8 ± 13.7^#^	N/A

Mean ± SD

^+^Blinded scores (excludes rigidity)

^#^At the 24-month study visit

^^^At the long-term follow-up assessment (5.4 ± 2.0 years after surgery)

### External validation of the motor progression sweet spot and connectivity model in an independent PD cohort

**Fig. 2:**
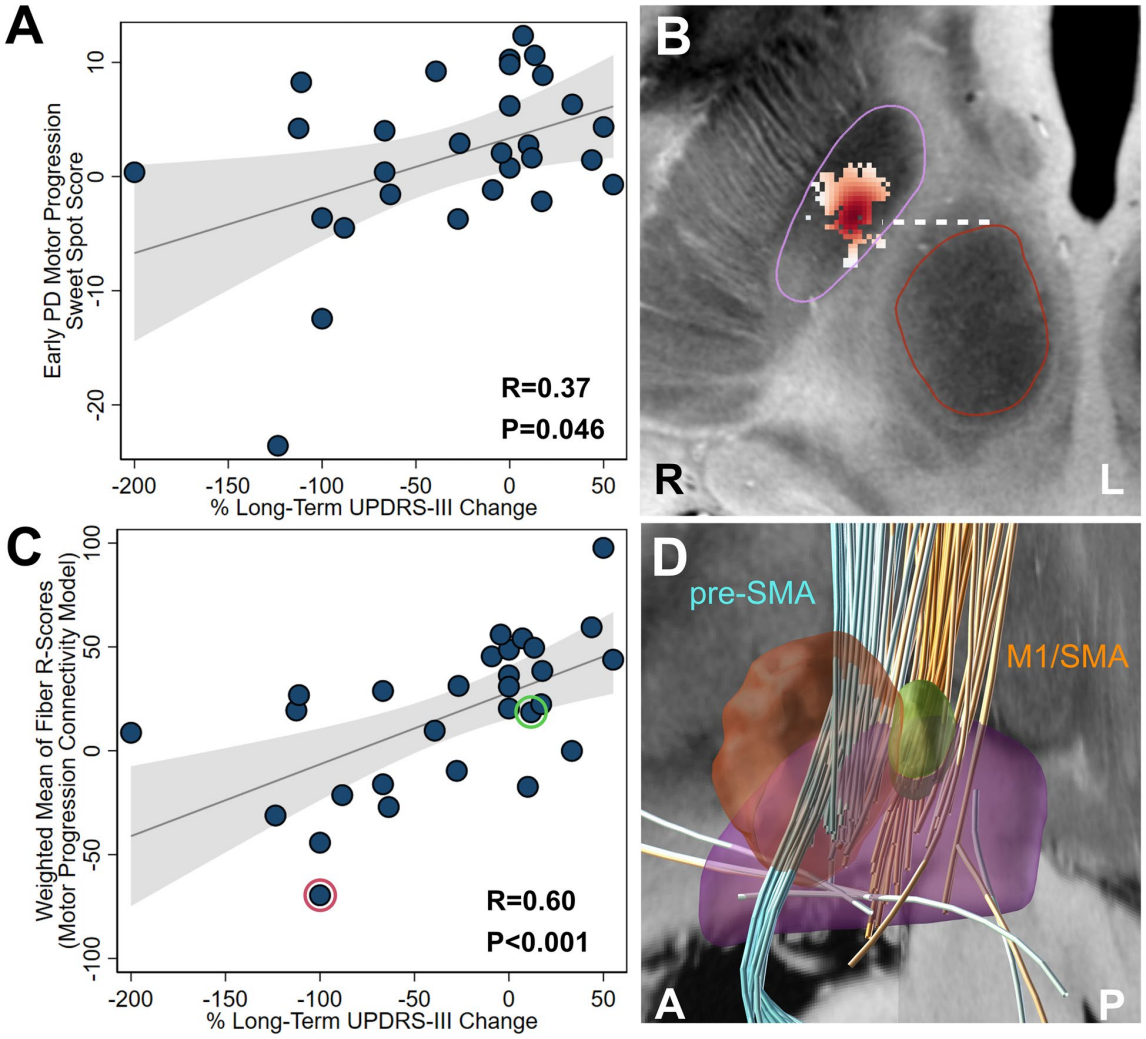
Evaluating the early PD motor progression sweet spot and connectivity model in advanced-stage PD. The motor progression sweet spot (**A**–**B**) and connectivity model (**C**–**D**) identified in 14 early-stage PD patients [1] were each used to estimate long-term motor outcomes in an independent cohort of 29 standard of care PD patients. **A** The degree of overlap with the motor progression sweet spot identified from the early DBS cohort (i.e., “Sweet Spot Score”) significantly correlated with long-term UPDRS-III improvements (DBS duration at follow-up = 5.4 ± 2.0 years; Spearman’s R = 0.37, P = 0.046). **B** The motor progression sweet spot from [1]. STN is outlined in purple. Red nucleus is outlined in red. Bejanni line = white dashed line [27]. **C** The degree of stimulating positive tracts and not stimulating negative tracts identified from the early DBS cohort (i.e., the “Weighted Mean of Fiber R-Scores” of the Motor Progression Connectivity Model) significantly correlated with long-term UPDRS-III improvements (Spearman’s R = 0.60, P < 0.001). Two illustrative example patients are marked with colored circles in panel C and their stimulation volumes are shown in panel D (top responder example = green; poor responder example = red). **D** The motor progression connectivity model [1] consists of positive (orange) tracts originating from M1 and SMA and negative (cyan) tracts originating from pre-SMA and cerebellum

Based on similarities between the sweet spot and connectivity associated with slower motor progression in early-stage PD and the STN region and connectivity associated with optimal motor improvement in standard of care PD patients, we hypothesized that the early-stage PD locations could be used to explain significant amounts of variance in long-term motor improvements in advanced-stage PD. To test this hypothesis, we conducted a new analysis using an independent cohort of 29 advanced-stage PD patients who received DBS as standard care (25/29 male; aged 60.1 ± 9.1 years at the time of surgery; Table 1). This new analysis assessed the ability of the early-stage PD motor progression sweet spot and connectivity models [1] to estimate long-term motor improvements, thereby extending our previous findings to a broader patient population. Mean disease duration at the time of DBS surgery for this cohort was 9.9 ± 5.0 years, and mean time from surgery to post-operative follow-up was 5.4 ± 2.0 years. The advanced-stage PD cohort received stimulation at an amplitude of 2.9 ± 0.9 V (n = 21) and 3.0 ± 0.6 mA (n = 8), with a pulse width of 60–90 µseconds and a frequency of 130 Hz. Long-term motor improvement was associated with optimal stimulation of the motor progression sweet spot in this independent cohort of advanced-stage PD patients (R = 0.37, P = 0.046; Fig. 2A, B). Optimal stimulation of the fiber tracts identified in the motor progression connectivity model also significantly associated with long-term motor improvement (R = 0.60, P < 0.001; Fig. 2C, D).

## Discussion

There are three main findings in this study. First, we revisited the DBS in early-stage PD pilot clinical trial dataset to evaluate active contact locations at various thresholds to associate optimal stimulation location with the degree of motor progression on a single subject level. This provides further evidence to suggest that proximity to a specific site within the dorsolateral STN is associated with slower motor progression [1]. Second, motor progression in the patients from the early DBS trial that were stimulated outside of the dorsolateral STN appeared to follow a similar course of motor progression to that of control subjects that received standard medical therapy in the trial. Finally, the motor progression sweet spot and connectivity models explained significant amounts of variance in long-term changes in motor symptoms in an independent group of advanced-stage PD patients.

Numerous studies have shown that stimulating a specific site within the dorsolateral STN associates with optimal short-term motor improvement (e.g., 6–12 months after surgery) in advanced-stage PD [3, 4, 11]. These studies used advanced statistical mapping to establish the relationship between proximity to the dorsolateral STN and symptomatic motor improvements [3, 4, 11]. The sites identified in these studies are remarkably similar to one another [19] and seem to correspond to the same site associated with slower motor progression and symptomatic motor benefit two years after surgery in early-stage PD [1]. Regarding brain connectivity, our previous study suggests that the optimal site receives cortical input from M1 and SMA [1]. Critically, input from pre-SMA was instead negatively associated with slower motor progression. Here, we demonstrated that the same site – as identified based on slower motor progression – also accounted for long-term motor improvements in an independent typical advanced-stage (standard of care) PD cohort. This suggests that the site associated with slower motor progression is equally suited to maximize long-term motor improvements following STN-DBS for PD. While the mechanisms underlying the potential association between stimulation of this STN region and slower motor progression remain unclear, several factors may contribute to this observed relationship. These could include long-term plasticity within the sensorimotor network [20, 21] and increased brain-derived neurotrophic factor (BDNF) signaling following STN stimulation [22].

The identified optimal stimulation site is based on a small sample size, which is a key limitation. Unfortunately, additional cohorts that measure motor progression following DBS in humans do not yet exist, so the site may not be readily validated using additional data. However, very similar optimal stimulation sites have been described by others. Confirming utility of this stimulation site to estimate long-term motor improvements in an independent larger cohort may add further credibility. Although rigidity could not be assessed due to the use of blinded videotape scoring of motor symptoms, video-based ratings of the UPDRS-III have been validated as a reliable measure [23]. To address this limitation in the early DBS study [1], we conducted a sensitivity analysis using unblinded rigidity scores with the blinded UPDRS-III motor score which resulted in a similar connectivity profile to our primary analysis and also yielded significant correlations. While this study focused on the relationships between stimulation location and motor outcomes, it is important to acknowledge that STN-DBS has been shown to impact a range of non-motor symptoms [24–26]. Future research should explore the potential influence of stimulation site on these important clinical outcomes. Finally, both cohorts were predominantly male, which limits the generalizability of the results to the broader PD population undergoing STN-DBS surgery and precluded the ability to evaluate for sex effects within the cohorts.

## Conclusion

While the association of this dorsolateral STN stimulation site with slower motor progression needs to be further validated by additional prospective studies, our results explore the current data at hand and conclude that the same site may be associated with both slower motor progression and long-term motor benefit with STN-DBS in Parkinson’s disease. The FDA has approved the conduct of a prospective, randomized, double-blind pivotal clinical trial evaluating safety and efficacy of DBS in early-stage PD (IDEG050016).
